# Patients' perception of user involvement in psychiatric outpatient treatment: Associations with patient characteristics and satisfaction

**DOI:** 10.1111/hex.13132

**Published:** 2020-09-16

**Authors:** Jens C. Thimm, Liss Antonsen, Wenche Malmedal

**Affiliations:** ^1^ Centre for Crisis Psychology University of Bergen Bergen Norway; ^2^ Department of Psychology Faculty of Health Sciences UiT The Arctic University of Norway Tromsø Norway; ^3^ Psychiatric Centre Helgeland Hospital Trust Mo i Rana Mo i Rana Norway; ^4^ Department of Public Health and Nursing Norwegian University of Science and Technology Trondheim Norway

**Keywords:** mental health, outpatient treatment, patient satisfaction, psychiatry, shared decision making, user involvement

## Abstract

**Background:**

The patient's right to be involved in treatment decisions is anchored in guidelines and legislation in many countries. Previous research suggests challenges in the implementation of user involvement across different areas of health care, including mental health. However, little is known about psychiatric outpatients’ experiences of being involved in their treatment.

**Objective:**

To investigate how psychiatric outpatients after treatment rate the degree to which they were included in the treatment and explore the associations between perceived user involvement, demographic characteristics of the sample and patient satisfaction.

**Design:**

Cross‐sectional.

**Setting and participants:**

The sample consisted of 188 psychiatric outpatients (67% female, mean age 42.2 years) who were discharged in the two years prior to data collection.

**Main variables studied:**

Perceived user involvement in psychiatric outpatient treatment and patient satisfaction as measured by the Psychiatric Out‐Patient Experiences Questionnaire.

**Results:**

About half of the participants rated the overall degree of involvement in their treatment as high or very high. The lowest percentage of participants reporting high or very high involvement was found for sufficient information to contribute to treatment decisions (36%). Female gender, higher education and, to a small degree, younger age were associated with more involvement. Perceived user involvement was strongly associated with treatment satisfaction.

**Discussion and conclusion:**

The findings suggest that user involvement in psychiatric outpatient treatment can be improved. Patient information that facilitates user involvement should be given more attention.

**Patient or Public Contribution:**

The hospital's user panel was involved in the development of items assessing user involvement.

## 1 INTRODUCTION

Promoted by the World Health Organization and anchored in the legislation of many Western countries, user involvement is emphasized in the provision of contemporary mental health care.^1^, ^2^ This emphasis marks a shift from paternalistic to partnership‐oriented models of the relationship between the health‐care provider and the patient, in which both parts contribute with their expertise and experience on equal terms to achieve desired outcomes.^3^, ^4^

In Norway, user involvement emerged as a central concept in health policy documents in the 1990s. In these documents, it is argued that the inclusion of the users’ experiences and knowledge is a value in itself and that it has a therapeutic effect and improves the quality of health services.^5^ In the Patient Right Act^6^ and the Specialized Health Care Act,^7^ among others, the patient's right to be involved in treatment decisions and the health‐care service's obligation to include patients in individual treatment planning and service evaluation are legally defined.

Although it is widely acknowledged that user involvement is an integral part of providing mental health‐care services, consensus about the definition of user involvement has not been reached. A variety of definitions of user involvement in mental health care has been suggested in the research literature.^8^ For example, Tambuyzer et al^9^ identified participation in decision making, active character of involvement (as opposed to being a passive recipient of information), involvement in a diverse range of activities (eg treatment, evaluation, research, training), expertise by lived experience and collaboration with professionals as key elements of user involvement in mental health. Similarly, Millar et al^8^ proposed that user involvement in mental health care has five defining attributes: a person‐centred approach, informed decision making, advocacy, obtaining service user views and feedback, and working in partnership. Accordingly, Millar et al defined user involvement in mental health services as 'an active partnership between service users and mental health professionals in decision making regarding the planning, implementation and evaluation of mental health policy, services, education, training and research. This partnership employs a person‐centered approach, with bidirectional information flow, power sharing and access to advocacy at a personal, service and/or societal level'.^8^ As stated in this definition, user involvement can occur in different areas and on different levels. Tambuyzer et al^9^ suggested four organizational levels in which user involvement is relevant: the individual patient (micro level), the health‐care service or the institution (meso level), mental health politics (macro level), and training and research (meta level).

However, although user involvement has become a legal standard internationally, research suggests that user involvement in mental health care is currently not fully realized in many countries,^10^, ^11^ including Norway.^12^ Bee et al^10^ identified poor information exchange between the health service and the user as the main cause for the lack of user involvement. Obviously, mental health professionals have a central role in facilitating user involvement. In general, mental health professionals have positive attitudes towards user involvement.^13^, ^14^, ^15^, ^16^ However, differences between professional groups have been noted, with social workers and psychiatric nurses tending to be more open to user involvement in mental health care than psychiatrists and psychologists.^17^ In addition, professionals working in outpatient care have shown more positive attitudes towards user involvement than professionals working in inpatient care.^14^ Lack of insight, difficulties with collaboration during episodes of severe mental illness, limited availability of the user's preferences and attitudes within mental health services perceived as disempowering the staff and users have been reported as potential obstacles to user involvement by professionals.^16^, ^18^, ^19^, ^20^

Despite slow progress in implementing user involvement on the organizational level and expressed concerns by service users about tokenism,^21^ study findings suggest that patients are generally interested in being involved in mental health services,^13^, ^22^ particularly with regard to their own treatment.^23^, ^24^ On the other hand, it has been observed that patients with severe mental illness preferred a more passive role in medical decision making than non‐psychiatric controls.^25^ However, although benefits of user involvement in mental health care for the individual patient are commonly assumed, research into the associations of user involvement with treatment outcomes on the individual level is scarce and inconclusive.^26^ For example, in Omeni et al's study,^13^ service providers and service users reported a positive impact of user involvement activities on self‐esteem and recovery. Tambuyzer and Van Audenhove^27^ found that user involvement is associated with patient satisfaction and increased empowerment. Nevertheless, despite encouraging findings, the evidence base is currently weak.^26^, ^28^

Most research on user involvement in mental health services has been conducted in inpatient settings, and little is known about the experiences of outpatients when it comes to participation in treatment decisions. The present study therefore aimed to investigate how psychiatric outpatients, after treatment, rate the degree to which they were included in different aspects of the treatment and whether perceived user involvement is related to the demographic background and the kind of treatment they had received. A second goal of the current investigation was to examine the associations between user involvement and treatment satisfaction in terms of the quality of clinical interaction, information provision and treatment outcome.

## 2 METHODS

### 2.1 Participants and procedures

Psychiatric outpatients who were discharged from treatment at the Psychiatric Centre of the Helgeland Hospital Trust in Mo i Rana in Norway in the two years prior to data collection in May 2017 were invited to participate in the present investigation. Patients were discharged because they finished treatment or dropped out. We approached patients who were discharged instead of patients who were currently in treatment because the former group had experienced the complete course of therapy and was therefore able to answer questions about user involvement in all phases of treatment, including termination, which we deemed an important area for user involvement in mental health care. A total of 1048 eligible patients were identified using the clinics electronic health record system. Mail addresses were lacking for 13 patients. Thus, the questionnaire containing the study measures was mailed to 1035 patients. In an accompanying letter, the recipients were informed about the purpose of the study, the researchers, voluntary participation and how to provide informed consent (by returning the questionnaire to the researchers). Participants were asked for permission to obtain their diagnosis from the electronic records. One reminder was sent after approximately three weeks. One hundred and eighty‐nine patients returned the questionnaire, which corresponds to a response rate of 18.3%. One participant reported in the comment field at the end of the questionnaire to never have been in treatment at the outpatient clinic and was thus excluded from the analyses. The final sample comprised 188 participants (67% female) with a mean age of 42.2 years (SD = 14.8 years, range 19 to 84 years). Additional demographic and clinical information about the sample is provided in Table 1.

**TABLE 1:** Demographic and clinical characteristics of the sample

Variable	Mean (SD)
Age	42.2 (14.8)
	N (%)
Gender	
Female	126 (67.0)
Male	59 (31.4)
Number of sessions	
One	7 (3.7)
2‐5	28 (14.9)
6‐12	45 (23.9)
More than 12	100 (53.2)
Treatment mode	
Medication	68 (36.2)
Individual sessions	173 (92.0)
Group sessions	52 (27.7)
Sessions with family members	19 (10.1)
Training programme to cope with symptoms	33 (17.6)
Marital status	
Married	53 (28.2)
Cohabitating	46 (24.5)
Neither married nor cohabitating	86 (45.7)
Educational level	
Primary education	43 (22.9)
Upper secondary education	77 (41.0)
College/university less than 4 y	53 (28.2)
College/university 4 y or more	11 (5.9)
First language	
Norwegian	178 (94.7)
Sami	0 (0)
Another Nordic language	1 (0.5)
Another European language	2 (1.1)
Non‐European language	4 (2.1)
Housing situation	
Live alone	57 (30.3)
Live with children	51 (27.1)
Live with partner	97 (51.6)
Live with parents, siblings, others	12 (6.4)
Residential care home	4 (2.1)
Other	7 (3.7)
Employment status	
Working	75 (39.9)
On sick leave	9 (4.8)
Disability benefit recipient	45 (23.9)
Work assessment allowance	44 (23.4)
Student	14 (7.4)
Unemployed	10 (5.3)
Diagnosis (ICD‐10)	
Bipolar affective disorders (F31)	11 (5.9)
Depressive episode (F32)	18 (9.6)
Recurrent depressive disorder (F33)	20 (10.6)
Phobic anxiety disorders (F40)	13 (6.9)
Other anxiety disorders (F41)	12 (6.4)
Reaction to severe stress and adjustment disorders (F43)	23 (12.2)
Hyperkinetic disorders (F90)	10 (5.3)
Other diagnoses (eg mental and behavioural disorders due to psychoactive substance use, persistent mood disorders, somatoform disorders, eating disorders, personality disorders and general psychiatric examination)	65 (34.6)
No diagnosis	16 (8.5)

Note.

Due to missing data points and multiple responses, the summed values of a category may fall below or exceed *N* = 188 or 100%.

Prior to the study, ethical approval was applied for from the Regional Committee for Medical and Health Research. The committee decided that approval from this entity was not required for the present investigation. The Norwegian Data Protection Service (NSD) was notified about the study (ref. nr 50690).

### 2.2 Measures

User involvement in psychiatric outpatient treatment was assessed with six self‐report items that cover different aspects of psychiatric outpatient treatment: (a) overall user involvement in treatment; (b) a say in the treatment package; (c) evaluation of sessions during treatment; (d) formulation of treatment goals; (e) termination of treatment; and (f) sufficient information to contribute to treatment decisions. The items were rated on a 5‐point scale from 'not at all' (0) to 'to a very high degree' (4). Item 2 was taken from the Psychiatric Out‐Patient Experiences Questionnaire.^29^ The remaining five items were developed for the present investigation because we were not aware of an existing measure of user involvement in psychiatric outpatient treatment. The items were selected based on previous research on user experiences in mental health care^30^ and a national policy document on user involvement in mental health care.^5^ The user panel of the hospital was involved in the process of item development and commented on the questionnaire. This procedure ensured the face validity of the items. The six items were combined to form a user involvement scale. A confirmatory factor analysis was conducted in Mplus 8.4^31^ using WLSMV estimation to test for the unidimensionality of the scale. The Comparative Fit Index (CFI), the Tucker Lewis Index (TLI) and the Standardized Root Mean Square Residual (SRMR) were used to assess model fit. The results (CFI = 0.99, TLI = 0.98, SRMR = 0.03) supported the proposed unidimensionality of the scale when Hu and Bentler's^32^ cut‐off values for good fit (CFI and TLI ≥ 0.95, and SRMR ≤ 0.08) are applied. The internal consistency of the scale was high (Cronbach's alpha = 0.90).

Treatment satisfaction was assessed with the Psychiatric Out‐Patient Experiences Questionnaire (POPEQ).^29^ The POPEQ is a self‐report measure consisting of 11 items that are answered on a 5‐point scale from 'not at all' (0) to 'to a very high degree' (4). In addition to an overall experience score, three subscale scores can be obtained: quality of clinical interaction (six items), information provision (two items), and outcome of the treatment (three items). Adequate‐to‐excellent psychometric properties have been reported for the POPEQ.^29^, ^33^ In the present study, the item of the POPEQ that was used in the user involvement scale was omitted when the quality of clinical interaction scale and the POPEQ total score were calculated. In the current sample, the POPEQ scales showed high internal consistencies with Cronbach's alphas ranging from 0.83 (information provision) to 0.95 (total score).

### 2.3 Statistical analyses

To examine the participants’ perception of user involvement in their treatment, the means, standard deviations and frequencies of the response options for the six user involvement items were calculated. Mean item scores were obtained for the user involvement total score and the POPEQ scales. The associations between user involvement and the gender and age of the participants were investigated with t tests and bivariate correlations, respectively. To test the relationships between treatment mode and user involvement, t tests were conducted. The associations of education level with user involvement were investigated by means of ANOVAs. When collecting the data, it was aimed at recruiting at least 150 patients to be able to detect small‐to‐medium effects in the correlation analyses with a power of 0.80 and at a significance level of *P* < .05 based on Cohen's^34^ criteria. No a priori power analysis was performed for comparisons of demographic groups with respect to user involvement. The final sample size of N = 188 exceeded the goal of 150 participants. A power analysis conducted with the pwr package^35^ in R 3.6.2^36^ showed that the group sizes were sufficient to detect medium effects (power = 0.80, *α* = 0.05) when examining perceived user involvement in relation to gender, educational level (provided that the two groups with university/college education were combined) and treatment mode (except for individual treatment). However, the number of participants in the different diagnostic categories was too small for an examination of the relationship between user involvement and diagnosis (ie only very large effects could have been detected with a power of 0.80 and an *α* of 0.05). Because the number of missing data points (4.5%) was within the range where multiple imputation provides negligible benefit,^37^ missing data were not replaced. Scale scores were computed for the user involvement scale, as were the POPEQ total and quality of clinical interaction scales when at least 80% of the items were answered. Due to the small number of items that constitute the POPEQ information provision (2 items) and outcome (3 items) scales, all items had to be answered in order to be included in the analyses. Computation of Cronbach's alpha, descriptive statistics, correlations and comparisons of group means were performed in IBM SPSS Statistics 26.

## 3 RESULTS

The means, standard deviations, and response frequencies of the six user involvement items are displayed in Table 2. Results showed that 54.8% of the participants rated the overall degree of involvement in their treatment as 'high' or 'very high'. For user involvement in the specific elements of outpatient treatment, the percentages of perceived involvement for 'to a high degree' or 'to a very high degree' were 47.3% for formulation of treatment goals, 45.3% for a say in the treatment package, 43.1% for evaluation of sessions, 36.7% for termination of treatment, and 35.6% for sufficient information to participate in decision making.

**TABLE 2:** Means, standard deviations and response frequencies of the user involvement items

Item	M	SD	Frequencies^a^ (%)				
			Not at all	To a small degree	To some degree	To a high degree	To a very high degree
Overall user involvement	2.47	0.99	4.3	11.7	27.1	43.6	11.2
A say in the treatment package	2.36	1.10	6.9	11.7	31.9	30.9	14.4
Evaluation of sessions	2.23	1.20	10.1	14.9	27.7	28.7	14.4
Formulation of treatment goals	2.32	1.15	7.4	16.0	25.0	33.5	13.8
Termination of treatment	2.05	1.30	17.0	12.8	29.3	21.8	14.9
Sufficient information	2.13	1.10	8.0	18.1	34.6	25.0	10.6

^a^ Due to missing values, the rows do not add to 100%.

The mean of the total score for the user involvement scale was 2.27 (SD = 0.94; range 0–4). With respect to the associations between the demographic characteristics of the sample and the user involvement scale score, there was a significant negative association with age (*r* = −0.16, *P* = .034). Women (M = 2.38, SD = 0.88) reported a higher degree of user involvement than men (M = 2.00, SD = 0.99, *t*(177) = −2.62, *P* = .010, *d* = 0.41). Educational level was related to user involvement, *F*(2, 175) = 6.75, *P* = .001, *η* ^2^ = 0.07. Post hoc tests (LSD) revealed that participants with primary education had lower scores on the user involvement scale (M = 1.82, SD = 1.08) compared to participants with college/university education (M = 2.46, SD = 0.85) and upper secondary education (M = 2.35, SD = 0.85) (all *P*s < 0.01).

The results of the analyses that tested the associations between treatment mode and user involvement are shown in Table 3. The only treatment mode that was significantly related to user involvement was a training programme to cope with symptoms. The association was positive.

**TABLE 3:** Results of t tests examining the associations between treatment mode and user involvement

Treatment mode	*M*	SD	*t*	*P*	*d*
Medication	2.36	0.84	1.01	.315	0.16
No medication	2.21	0.99			
Group sessions	2.32	0.87	0.46	.646	0.07
No group sessions	2.25	0.97			
Sessions with family members	2.22	1.00	−0.23	.818	0.05
No sessions with family members	2.27	0.93			
Training programme to cope with symptoms	2.64	0.61	3.45^a^	>.001	0.50
No training programme to cope with symptoms	2.18	0.98			

^a^ Because Levene's test for equality of variances was significant (*P* = .011), the t‐value for unequal variances is reported.

The means, standard deviations, and correlations of the POPEQ scales with the user involvement scale are displayed in Table 4. Correlation analyses showed that the user involvement scale was highly correlated with the POPEQ scales ranging from 0.74 (outcome of the treatment) to 0.84 (total score) (all *P*s < .001).

**TABLE 4:** Descriptives of the POPEQ scales and correlations with the user involvement scale total score

POPEQ scale	*M*	SD	*r*
Total score	2.69	0.94	0.84*
Quality of clinical interaction	2.70	0.96	0.80*
Information provision	2.39	1.19	0.77*
Outcome of the treatment	2.80	1.02	0.74*

* *P* < .001.

## 4 DISCUSSION

The aim of the current study was to investigate psychiatric outpatients’ perceptions of user involvement after ended treatment, as well as demographics and treatment‐related predictors of user involvement and the associations of user involvement with treatment satisfaction. Results showed that more than half the participants reported their overall involvement in their treatment at the outpatient clinic as high or very high. Ratings for user involvement in the specific components of treatment were lower than for the perceived overall involvement. A scale based on the user involvement items showed close associations with user satisfaction regarding the quality of the interactions with the clinicians, provision of information and treatment outcome.

In retrospect, a majority of participants (54.8%) rated the overall user involvement in their treatment as high or very high. On the other hand, this result means that nearly half the participants experienced that they had not at all or, only to some degree, been involved in important treatment decisions. This finding is line with previous research^10^, ^11^ and shows that there is room for improvement regarding user involvement in psychiatric outpatient treatment despite its emphasis in legal regulations and guidelines. With respect to specific areas of user involvement in outpatient treatment, the highest percentage of participants who rated their involvement as high or very high concerned the formulation of treatment goals (47.3%). The formulation of specific and tangible goals is especially emphasized in psychological treatments compared with other therapeutic approaches (eg medication). Collaboration and consensus on treatment goals is an important part of the psychotherapeutic alliance,^38^ and has been shown to enhance treatment outcomes.^39^, ^40^ As such, agreement on goals is emphasized in different psychotherapy approaches.^41^ Since most patients in the sample received a form of psychological treatment, the involvement of patients in the formulation of treatment goals in the present investigation must be considered low. It can further be argued that the theoretical underpinnings of a treatment for psychiatric disorders may influence the level of user involvement and that the critical examination of the value base of a given approach can contribute to increase user involvement.

Notably, the least endorsed item was sufficient information to participate in decision making with 35.6% of participants rating this aspect of user involvement as high or very high. Information is highly prioritized by patients receiving treatment in medical^42^ and mental health settings..^43^, ^44^, ^45^ However, patients’ need for information is often not met.^46^, ^47^ Crucially, adequate information is essential for user involvement.^10^ The finding of the current study that only a third of the participants reported that they had received sufficient information to participate in treatment decisions suggests an urgent need to improve the information given to the patients. Providing decision aids, that is materials, media or interventions that are designed to support patients in the decision‐making process^48^ rather than general information about diagnosis and treatment, has shown to be beneficial in terms of increased patient knowledge, improved patient‐clinician communication, and decreased decision conflict, indecision about personal values and passivity.^49^ In addition to information, Joseph‐Williams et al^50^ argue that the experience of power imbalance in the relationship between the clinician and service user needs to be addressed to achieve meaningful user involvement.

In the current sample, the endorsement of involvement in the termination of treatment was almost as low as for sufficient information to participate in decision making, indicating that the participants perceive that the decision about ending the treatment is often made by the mental health professional. The assumption that the treatment goals are attained in addition to large caseloads may be barriers to clinicians involving service users in this decision. Although patients often agree on termination initiated by the clinician,^51^ research suggests that participation in the termination process is appreciated by patients and related to experiences of good and productive final sessions^52^ and a stronger therapeutic bond and higher satisfaction.^51^

Results further showed associations of the participants' age, gender and educational level with perceived user involvement, where younger, female and more highly educated patients report more involvement. The effects of gender and education were medium‐sized, whereas the effect of age was small based on common criteria.^34^ Previous studies suggest that young, female and educated patients prefer a more active role in psychiatric treatment,^45^, ^53^ which is in line with the current findings. With regard to treatment mode, only training to cope with symptoms was related to user involvement, where patients who received this treatment experienced more user involvement than those who did not. Typically, in a training programme, the therapist and the patient collaborate on designing exercises that are therapeutic and feasible to achieve a specific goal (eg anxiety reduction in a phobic situation). Thus, user involvement in this treatment element is usually already firmly established, resulting in higher user involvement ratings in the present study. Other treatment modes, by contrast, do not differ from each other when it comes to perceived user involvement.

The perception of user involvement was strongly connected with treatment satisfaction. High correlations suggest that patients who experience a high degree of user involvement also tend to rate the quality of the interaction with the clinician more favourably, are more satisfied with information and have better outcomes. Previous studies have demonstrated a relationship between user involvement and patient satisfaction.^54^ Findings regarding the associations between user involvement and treatment outcomes in psychiatric samples are currently inconclusive. There has been noted, however, an association when self‐report is used and affective‐cognitive outcomes are assessed.^28^ The current findings align with this observation.

Taken together, the results of the current study show that user involvement in psychiatric outpatient treatment can be improved. Interventions aiming at enhancing user involvement should target patients, professionals and organizations. The integration of user involvement into routine outcome measuring has shown promising preliminary results in terms of less conflicts and better outcomes in individuals with depression.^55^ In this effort, it has been suggested that drawing on knowledge from implementation science^56^ and established models of factors should be taken into account when implementing health innovations (eg implementation climate, implementation planning and individual characteristics of service providers).^57^ More research is needed that focuses on identifying barriers to user involvement in the specific aspects of psychiatric outpatient treatment on the part of the patients and the clinicians as well on the organizational level. Future research should also develop and evaluate interventions and aids to facilitate user involvement that are specifically tailored to the characteristics of psychiatric outpatient treatment.

The present study has some limitations that must be considered when interpreting the results. Importantly, causal relationships cannot be inferred from the results due to the observational and correlational design of the study. For example, it cannot be concluded that user involvement has a direct effect on treatment outcome. Further, participants were recruited from only one outpatient clinic, and the generalizability to other outpatient health services is unclear. The generalizability of the current findings is also limited by a relatively low response rate. It was thus not possible to determine how representative the sample was for the population of the clinic. The response rate would probably have been higher if patients were asked for participation in the study at discharge. Unfortunately, this was not possible due to limited time for the data collection. The recruitment of patients who had finished treatment ensured that the participants were able to report on their experiences of user involvement for the whole course of treatment. On the other hand, we do not know how long‐term users of the clinic are experiencing their involvement. In addition, recall biases can have affected the responses. It is possible that patients who were very satisfied or very dissatisfied with the treatment were more likely to participate than those in between. Similarly, patients’ willingness to participate in the investigation can have been influenced by whether discharge was due to treatment completion or their decision to terminate the treatment. The sample was also highly heterogeneous with respect to diagnoses, resulting in relatively few participants who shared the same diagnoses. It was therefore not possible to examine the associations between diagnosis and perceived user involvement in the sample. User involvement was assessed with a scale that was constructed for this investigation, and its psychometric properties have not been previously established. Patients’ preferences with regard to user involvement were not measured. The training of the mental health professionals was not assessed, and the associations between perceived user involvement and therapist training could therefore not be examined.

In conclusion, the findings of the present study show a moderate degree of perceived user involvement in psychiatric outpatient treatment. Female gender, higher education and, to a small degree, younger age were associated with more user involvement. Perceived user involvement was strongly associated with treatment satisfaction.

## Acknowledgements

We would like to thank Cecilie Tronstad for her help with the data collection. We also thank the people who took part in the study.

## Data Availability

The data that support the findings of this study are available from the corresponding author upon reasonable request.
